# Multiple Protein Biomarker Assessment for Recombinant Bovine Somatotropin (rbST) Abuse in Cattle

**DOI:** 10.1371/journal.pone.0052917

**Published:** 2012-12-27

**Authors:** Susann K. J. Ludwig, Nathalie G. E. Smits, Grishja van der Veer, Maria G. E. G. Bremer, Michel W. F. Nielen

**Affiliations:** 1 RIKILT-Institute of Food Safety, Wageningen UR, Wageningen, The Netherlands; 2 Laboratory of Organic Chemistry, Wageningen University, Wageningen, The Netherlands; Moffitt Cancer Center, United States of America

## Abstract

Biomarker profiling, as a rapid screening approach for detection of hormone abuse, requires well selected candidate biomarkers and a thorough *in vivo* biomarker evaluation as previously done for detection of growth hormone doping in athletes. The bovine equivalent of growth hormone, called recombinant bovine somatotropin (rbST) is (il)legally administered to enhance milk production in dairy cows. In this study, first a generic sample pre-treatment and 4-plex flow cytometric immunoassay (FCIA) were developed for simultaneous measurement of four candidate biomarkers selected from literature: insulin-like growth factor 1 (IGF-1), its binding protein 2 (IGFBP2), osteocalcin and endogenously produced antibodies against rbST. Next, bovine serum samples from two extensive controlled rbST animal treatment studies were used for *in vivo* validation and biomarker evaluation. Finally, advanced statistic tools were tested for the assessment of biomarker combination quality aiming to correctly identify rbST-treated animals. The statistical prediction tool k-nearest neighbours using a combination of the biomarkers osteocalcin and endogenously produced antibodies against rbST proved to be very reliable and correctly predicted 95% of the treated samples starting from the second rbST injection until the end of the treatment period and even thereafter. With the same biomarker combination, only 12% of untreated animals appeared false-positive. This reliability meets the requirements of Commission Decision 2002/657/EC for screening methods in veterinary control. From the results of this multidisciplinary study, it is concluded that the osteocalcin – anti-rbST-antibodies combination represent fit-for-purpose biomarkers for screening of rbST abuse in dairy cattle and can be reliably measured in both the developed 4-plex FCIA as well as in a cost-effective 2-plex microsphere-based binding assay. This screening method can be incorporated in routine veterinary monitoring programmes: in the European Union for detection of rbST abuse and in the control of rbST-free dairy farms in the United States of America and other countries.

## Introduction

Many different techniques are available for detection of hormone abuse in sports doping and veterinary control, which all have to fulfil the requirements to be reliable, comparably fast and affordable. Biomarker profiling was suggested as a rapid screening approach for detection of doping practices because of its many advantages over the direct detection of the particular abused substances [1]. Biomarker profiles are indicative for more than one administered agent as they reflect the physiological effect, hence, the abuse of unknown compounds can also be detected [1], [2]. Furthermore, in many cases, the analysis of biomarker profiles enables the detection of abused substances for a longer time period, because the biological effect lasts longer than the abused substance itself can be detected in the body [3], [4]. A lot of work was focused on the identification of indicative biomarkers and the development of assays for detection of those [2], [5]–[10]. But the suitability and discriminative power of each biomarker has to be evaluated in controlled studies where a treated group is compared with an untreated one [11]–[13].

Extensive studies were done for the biomarker-based detection of recombinant somatotropin (ST; or growth hormone, GH) in sports doping, where ST is abused by athletes for their performance enhancement [14]–[18]. A similar screening approach can be chosen for the detection of recombinant bovine ST (rbST) abuse in dairy cattle, where the hormone is administered for enhanced milk production [19], [20]. The administration to dairy cattle is approved by the U.S. Food and Drug Administration in the United States of America and allowed in several other countries [21]. But treating cows with rbST is forbidden in the European Union since 1999 because of animal health and welfare reasons [22]. By European regulation, screening and confirmatory methods should be available for the detection of (ab)used veterinary drugs, with for screening, a maximum false-compliant rate of 5% (ß error) [23]. In contrast to the well-established human biomarker-based screening approach, the issue of rbST-dependent biomarker detection is still in its infancy: actually, routine veterinary control for rbST abuse has not been implemented at all, despite the EU ban. So far developed methods which detect rbST directly, such as immunoassays or mass spectrometry-based methods, suffer from the short half-life of rbST. Although biweekly injections containing slow-release formulations are used to prolong the presence of rbST in the cows' body, the protein levels in treated animals cannot be distinguished from the background level throughout the whole two-week inter-injection period and large inter-individual differences in blood rbST levels were reported [19], [20], [24]–[27]. Furthermore, rbST immunoassays were not capable to distinguish the almost identical recombinant and endogenous forms of bST [19], [20], [24], [25] and mass spectrometry-based methods on the other hand required very tedious sample preparation procedures [26], [27]. For screening of rbST in cattle, a few biomarker-based methods were developed, but focused on a single candidate biomarker only [4], [9], [28]–[30]. In a recent study, three candidate biomarkers were combined in one screening tool, but the <5% false-compliant rate target could not be achieved [31]. Nevertheless, biomarker-based screening for rbST can be considered a very promising start for detecting rbST abuse in dairy cows.

Biomarkers indicative for ST abuse are described in detail in literature and several of them are listed and referenced in Table 1. From these, we selected four different candidate biomarkers. These included two biomarkers of the IGF-1 axis, which respond quickly upon rbST treatment, namely insulin-like growth factor-1 (IGF-1) and IGF binding protein 2 (IGFBP2). The other two biomarkers were expected to show a delayed but long-lasting response; these are osteocalcin (marker of bone turnover) and antibodies which are endogenously produced against rbST (anti-rbST-antibodies).

**Table 1:** Candidate biomarkers for ST abuse and their expected response upon ST treatment in human and cows.

Biomarkers	response to ST	described for	reference
Acid labile subunit (ALS)	increase	human	[45]
Anti-rbST-antibodies	increase	bovine	[30], [31], [33], [34]
Apolipoprotein A-1 (APOA1)	decrease	human	[8]
C-terminal cross-linked telopeptide of collagen I (ICTP)	increase	human	[13], [45], [46]
C-terminal propeptide of procollagen I (PICP)	increase	human	[13]
Haemoglobin α-chain (HbA1)	increase	human	[6]
IGF binding protein 2 (IGFBP2)	decrease	bovine	[31], [47]
IGF binding protein 3 (IGFBP3)	increase	human	[45]
Insulin-like growth factor (IGF-1)	increase	human	[45], [46]
Inter-alpha-trypsin inhibitor heavy chain H4 (ITIH4)	decrease	human	[8]
Leucine-rich a-2-glycoprotein (LRG)	increase	human	[9]
N-terminal propeptide of procollagen I (PINP)	increase	human	[12], [45]
N-terminal propeptide of procollagen III (PIIINP)	increase	human	[12], [13], [45], [46]
Osteocalcin	increase	human	[12], [13]
Transthyretin (TTR)	increase	human	[8]
α-1 antitrypsin (AAT)	increase	human	[8]

**Figure 1:**
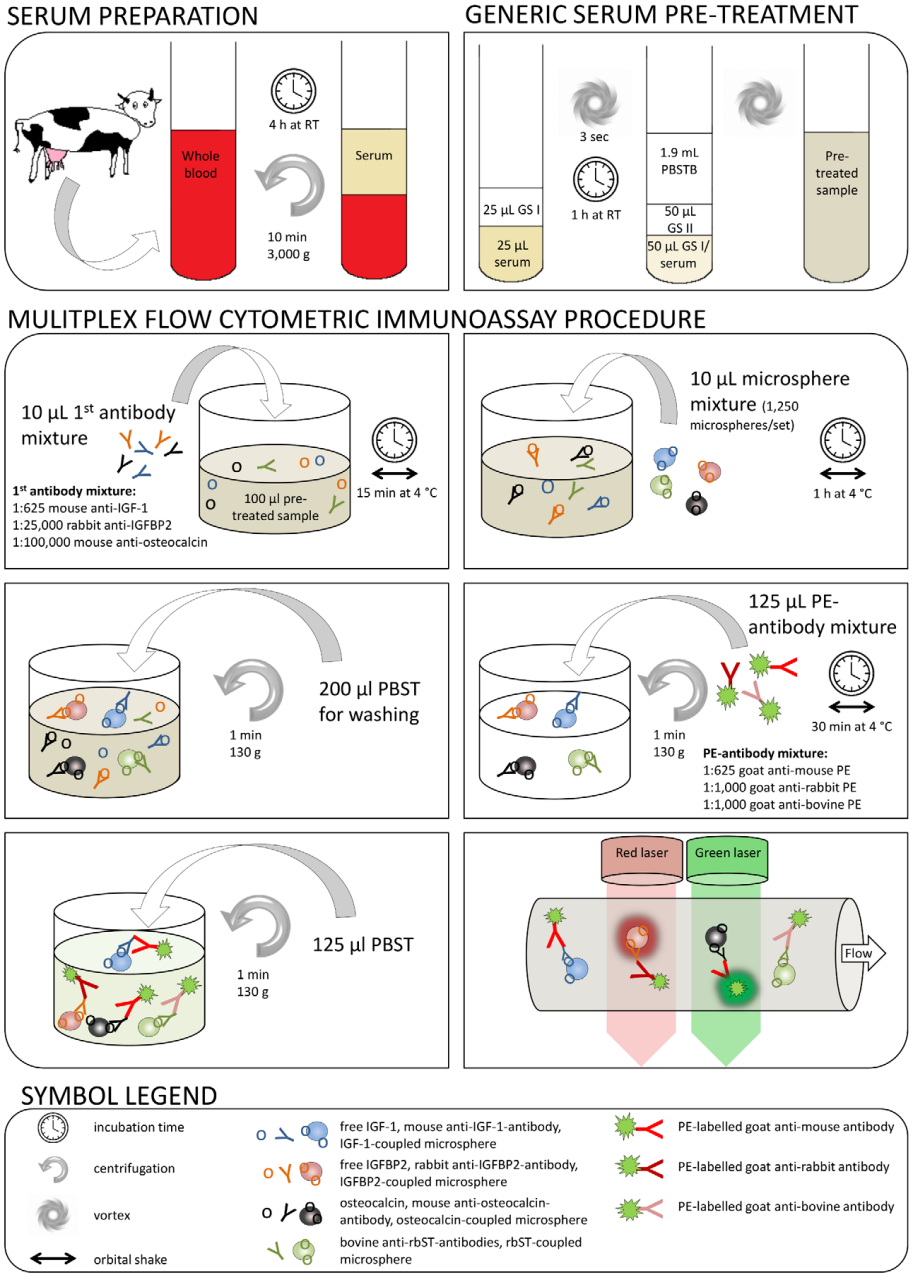
Work flow for serum preparation, generic serum pre-treatment and 4-plex FCIA for serum candidate biomarkers. A detailed description can be found in Materials S1. Abbreviations: h – hour, IGF-1 – insulin-like growth factor 1, IGFBP2 – IGF binding protein 2, GS I – glycine solution I, GS II glycine solution II, min – minutes, PBST – phosphate-buffered saline with 0.05% (v/v) Tween-20, PBSTB – 0.1% (m/v) BSA in PBST, PE – phycoerythrin fluorescent label, rbST – recombinant bovine somatotropin, RT – room temperature, sec – seconds.

To be able to screen for these four candidate biomarkers in serum, we developed a 4-plex flow cytometric immunoassay (FCIA) enabling parallel biomarker analysis in a single sample. For IGF-1, IGFBP2 and osteocalcin, a competitive inhibition assay format was chosen, where the respective candidate biomarker is covalently coupled to one set of colour-encoded microspheres. The different microsphere sets can be discriminated by a red laser (Figure 1). Biomarker-specific and generic fluorescent secondary antibodies are used for quantification with a green laser. Due to the inhibition format, high sample biomarker concentrations yield low fluorescence signals and *vice versa*. For the anti-rbST-antibodies, a direct assay format with an rbST-coupled colour-encoded microsphere set was used, where the anti-rbST-antibodies bind and can be detected by fluorescently labelled anti-bovine detection antibodies. Here, a high biomarker level leads to a high fluorescence signal. With this 4-plex FCIA, biomarker profiles were measured in serum samples. Based on the biomarker profiles of 67 untreated animals from different origins, we assessed the inter-individual and physiological variability of these biomarkers within dairy cattle and determined decision limits, beyond which a sample could be classified rbST-treated. Then, we used a large set of serum samples obtained from two independent controlled rbST animal treatment studies to evaluate the discriminative power of each candidate biomarker and of all combinations of biomarkers for distinguishing rbST-treated from untreated cows. Following thorough statistical evaluations, the value of individual and multiple biomarkers was assessed for the prediction of rbST abuse in dairy cows.

The overall aim of the study was the development and validation of a chemical analytical method for rbST-dependent biomarker detection according to European legislation for screening methods [23] and a data analysis approach for identifying biomarker combinations, which can reliably predict rbST abuse. This aim was reached with the help of a statistical prediction model based on the biomarker combination endogenously produced antibodies against rbST and osteocalcin.

## Results and Discussion

For the prediction of rbST abuse in dairy cows, we selected candidate biomarkers based on information found in literature (Table 1). These were markers of the IGF-axis (such as IGF-1 and IGFBP2) and bone markers (such as osteocalcin), known to be influenced by somatotropin and previously examined by the GH-2000 group for detecting somatotropin abuse in athletes [13], [32]. Furthermore, the immune response of cows treated with rbST was examined thoroughly and we used the presence of the specific endogenous antibodies against rbST as a biomarker for its abuse [4], [30], [33], [34]. Although PIIINP, a marker of collagen turnover, is known to show potential in human and bovine hormone abuse detection [10], [18], it has not been included into our biomarker panel yet because of the lack of a suitable commercially available standard protein and antibody.

### Development of a 4-plex flow cytometric immunoassay

For the simultaneous detection of these four candidate biomarkers, we developed a generic sample pre-treatment and 4-plex flow cytometric immunoassay (FCIA). To this end, our previously reported 3-plex assay [31] was extended with the biomarker osteocalcin. Adding osteocalcin to the existing triplex FCIA did not result in major interferences of any of the assay components of the four combined biomarker assays (data not shown). IGF-1 and osteocalcin concentrations of tested serum samples were calculated based on the obtained standard curves in serum-matched buffer (Figure S1). The 4-plex FCIA is capable of determining IGF-1 and osteocalcin concentrations in the relevant range in serum, namely 64–400 ng mL^−1^ for IGF-1 and 32–320 ng mL^−1^ for osteocalcin (note that serum samples were diluted 80-times prior to analysis, thus the standard curves cover protein concentrations of 0.8–5 ng mL^−1^ for IGF-1 and 0.4–4 ng mL^−1^ for osteocalcin). For IGFBP2, the standard protein could not completely inhibit the B0 signal; therefore, we decided to work with normalized responses (B/B0) for the data analysis. For the induced anti-rbST-antibodies, we worked with the responses normalized to a single standard serum (B/Bd).

The generic sample pre-treatment was necessary for releasing IGF-1 from its binding protein-complex and preventing non-specific binding in the detection of anti-rbST-antibodies. The rather harsh pre-treatment protocol did not affect the detection quality of osteocalcin, thus it could be adopted for the combined 4-plex FCIA. Note that adding IGF-2 in excess, as done in commercially available human IGF-1 immunoassays, improved neither the normalized standard curves nor the detection of biomarker level differences in between treated and untreated animals. The developed assay showed high reproducibility for all measured candidate biomarkers (Table 2) and a comparable sensitivity to previous single biomarker methods [35], [36]. However, the newly developed 4-plex FCIA has several advantages, such as the simultaneous measurement of all four markers in one sample from one well of a microtiter plate, which saves sample material, work load and time. Additionally, only one washing step was required compared to an average of six washing steps in a conventional enzyme-linked immunosorbent assay, making the 4-plex FCIA much faster and easy-to-use. The whole assay procedure, starting from a serum sample until the results from the flow cytometer for all four candidate biomarkers, takes 3.5 hours for a whole 96 well microtiter plate. This demonstrates that the 4-plex FCIA is a rapid and promising screening tool for the detection of the four candidate biomarkers in serum.

**Table 2:** 4-plex FCIA assay performance characteristics for the single candidate biomarkers.

	Candidate biomarkers			
Performance characteristics	IGF-1	IGFBP2	Anti-rbST antibodies	Osteocalcin
IC_50_	1.5^a^	9.5^a^	-	1.1^a^
Inter-assay variation	15.7^b^	7.9^b^	22.3^b^	17.1^b^
Intra-assay variation	6.4^b^	5.7^b^	9.4^b^	9.5^b^
Decision limit	216^a^	0.52^c^	1.62^d^	160^a^
Stability	The 4-plex FCIA can be performed stably over several months by different staff.			
Specificity	No unwanted interaction in between the assays (analytes and antibodies) observed.			

IC_50_ related to 80-times diluted samples.

a in ng mL^−1^.

b in %.

c of B/B0.

d of B/Bd.

### Single candidate biomarker profiles of untreated and of rbST-treated cows

After successful development of the 4-plex flow cytometric immunoassay, decision limits for each single candidate biomarker were calculated by analysis of sera from 67 untreated dairy cows (see paragraph 9.1 in the materials and methods section). Compared to the number of tested athletes in human studies, the number of tested control animals may seem to be rather low, but the variation within the dairy population is expected to be much lower, because of several reasons: First, only female cows have to be taken into account. Second, milking only occurs after first calving (usually at 20–24 months of age), thus after puberty, in which levels of IGF-1, IGFBP2 and osteocalcin are mainly changed due to growth and are more stable thereafter [37]–[39]. Third, since in this region of Europe mainly Frisian Holstein cows are used for milk production, we focussed on this particular race for the development of the test. And fourth, we do not need to consider sick animals, since their milk will not be allowed for consumption due to the presence of veterinary drug residues and therefore treatment with rbST is useless for sick dairy cows. Thus, the overall relative variation expected in dairy cows is anyway much lower than in athletes, where gender, different ethnicities, the effect of sports discipline, injury and all age groups need to be considered.

**Figure 2:**
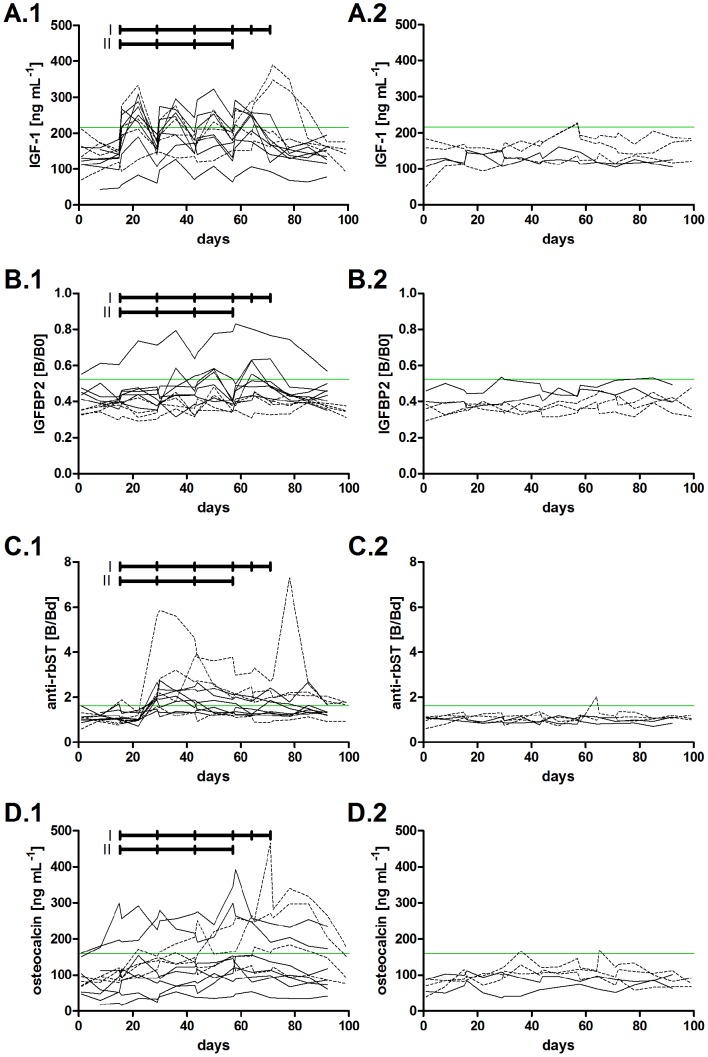
Biomarker profiles of rbST-treated (left) and untreated (right) dairy cows. Profiles from animal study I (dotted lines) and animal study II (solid lines) are shown. Sera from adaption period (3 sera from every cow), treatment period (13 sera per cow from animal study I and 9 sera per cow from animal study II) and withdrawal period (5 sera per cow from animal study I and 6 sera per cow from animal study II) were measured in duplicate. Biomarkers shown are concentrations of IGF-1 (A), B/B0 levels of IGFBP2 (B), B/Bd levels of antibodies against rbST (C) and concentrations of osteocalcin (D). The rbST treatment schedules for both animal studies are indicated by two black horizontal bars and decision limit per biomarker by the green horizontal line. Note that cows from animal study II received two additional rbST injections after the biweekly treatment period.

Decision limits were 216 ng mL^−1^ for IGF-1, 0.52 B/B0 for IGFBP2, 1.62 B/Bd for anti-rbST-antibodies and 160 ng mL^−1^ for osteocalcin and are shown as green horizontal lines in Figure 2. Results of samples exceeding this limit were considered positive.

Then, biomarker profiles of the dairy cows from both animal studies were measured (Figure 2). Results of the cows from animal study I are shown in dotted lines whereas the results of animal study II are shown in solid lines. Note that the animals from animal study I received two additional weekly rbST injections after the biweekly treatment period (the treatment schedules of both animal studies are indicated by the black horizontal bars above the graphs and shown in Figure S2).

IGF-1 levels were found to be elevated directly after rbST treatment (Figure 2A.1) and returned back to baseline before the next treatment. This short response time was observed before in human studies, where IGF-1 concentrations were back to baseline one week after termination of somatotropin treatment [32]. Nevertheless, in athletes, IGF-1 stayed elevated throughout the treatment period. This difference in IGF-1 response to somatotropin treatment could be due to the fact, that athletes were injected daily and, although a slow-release formulation was used in the here presented study, the biweekly treatment schedule does not reflect the same situation of permanently present somatotropin in circulation. IGF-1 levels of untreated animals (Figure 2A.2) remained below the decision limit. The found IGF-1 concentrations are consistent with previously reported serum IGF-1 concentrations in dairy cows [31], [40].

IGFBP2 levels (Figure 2B) are expected to decrease upon rbST treatment [32], [41]. The IGFBP2 assay is of an inhibition format, thus B/B0 levels are inversely correlated with the concentration. Hence, higher B/B0 levels are expected after rbST treatment. For some of the rbST-treated cows, a slight increase in B/B0 levels can be observed after treatment (Figure 2B.1) with a decrease to baseline before the next treatment. But this pattern is not as pronounced as for IGF-1. Furthermore, only occasionally a value exceeded the decision limit. Only the results of one cow were clearly above the decision limit, but these values were observed already during the adaptation period. In humans and despite large inter-individual differences, mean IGFBP2 levels responded quite well upon ST treatment, but the athletes were treated daily on three subsequent days [32]. B/B0 levels of untreated animals (Figure 2B.2) remained below the decision limit at almost all times.

For the antibodies, endogenously produced by the cow as an immunological response upon rbST treatment [30], a delayed increase in signal was observed (Figure 2C.1). Most of the cows developed antibodies approximately 2 weeks after the first rbST injection and a maximum in response could be seen around the third injection (four weeks after start of rbST treatment). Thereafter, the responses declined slowly. Zwickl *et al.* reported an increase of antibody formation within the first three months of rbST treatment and a decline thereafter, but the amount of rbST administered in their study was much higher than recommended by the manufacturer and applied here [34]. For the untreated cows in our studies (Figure 2C.2), only one result was found to be above the decision limit.

For osteocalcin, a slow increase in concentration was observed after rbST treatment (Figure 2D.1) compared to the untreated cows where the concentrations remained below the decision limit at almost all times. A similar effect on osteocalcin levels was observed in the human GH-2000 study [13]. Osteocalcin concentrations in our studies increased consistently in the 8 week treatment period, no gradual decline was observed as for the anti-rbST-antibodies. A slow osteocalcin decrease was noticed after rbST withdrawal but values remained above the decision limit for some of the cows until the end of the animal study.

For all of the candidate biomarkers large inter-individual physiological differences in biomarker levels were apparent as for example seen in the adaptation period of the treated animals. IGF-1, IGFBP2 and osteocalcin levels differed quite a lot between individual animals. Biomarker levels are known to be influenced by many factors such as age and state of lactation. Nevertheless, the expected variation is much smaller than in athletes tested for ST abuse as already discussed above. Note that we accounted for the variation in our untreated reference population used to assess the decision limits. Also the response upon rbST treatment differed in every individual cow. Some cows showed a big increase in IGF-1 levels short after injection while others did not show any response above decision limit (non-responders). Also for osteocalcin, some cows hardly showed any response after treatment.

**Figure 3:**
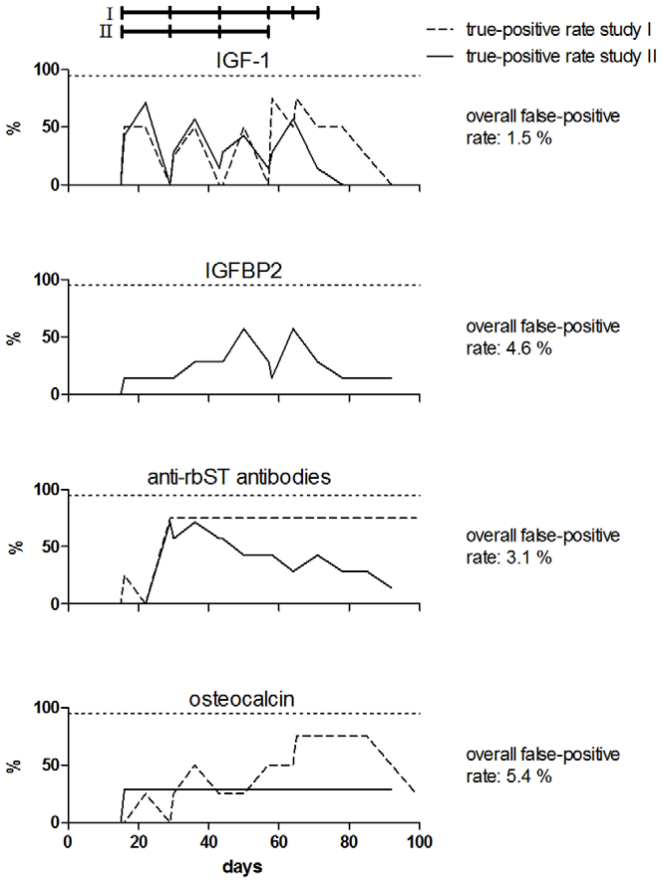
Predictive power of each single candidate biomarker for indicating rbST abuse. True-positive rates were calculated for all samples from rbST-treated cows in their treatment and withdrawal periods of study I and II. False-positive rates were calculated for all samples from untreated cows from the two animal studies (adaptation period samples from all cows and all the samples from untreated cows). Samples were considered positive if their biomarker value exceeded the respective decision limit. The treatment schedules of the two controlled animal studies are indicated by black horizontal bars on top of the graph. The targeted 95% true-positive (<5% false-compliant) rate according to 2002/657/EC is indicated by the dotted horizontal line.

The predictive power of each candidate biomarker was assessed by calculating true-positive rates for all samples from rbST-treated cows in their treatment and withdrawal period (Figure 3). False-positive rates were calculated from untreated cows during the whole animal experiment (adaptation period samples from all cows and all the samples from untreated cows). High true-positive rates were reached by IGF-1 already at the beginning of the treatment period. Similar response patterns were observed for both studies. Only the double injections in study I led to a changed IGF-1 pattern. Also for the anti-rbST-antibodies, high true-positive rates of 75% were seen after the second rbST injection. But the response was study-dependent: while the animals from study I (equal age of 5 years) were found positive after the second injection until the end of the study period, a gradual decrease of the number of positively found animals was observed in study II (age ranged from 2 to 8 years). This could be due to the different ages of the animals in study II. We saw that the antibody response tended to be higher in the older animals. Younger animals also showed antibody response, which declined more quickly than for the older animals. For osteocalcin, as already seen in Figure 2D.1, some of the rbST-treated cows did not show osteocalcin concentrations beyond the decision limit in both studies. The increase of true-positive found samples at the end of the treatment period in study I was due to the double rbST injections. As already expected from the biomarker profiles (Figure 2B.1), IGFBP2 did not show high true-positive rates, i.e., none of the animals from study I and only some animals in study II were found above the decision limit. For all of the candidate biomarkers, false-positive rates were quite low, indicating a high specificity of all of the biomarkers towards rbST treatment. Nevertheless, none of the candidate biomarkers reached the targeted 95% true-prediction (<5% false-compliant) rate at any time point required for a screening method according to Commission Decision 2002/657/EC [23].

### Additive biomarker analysis

**Figure 4:**
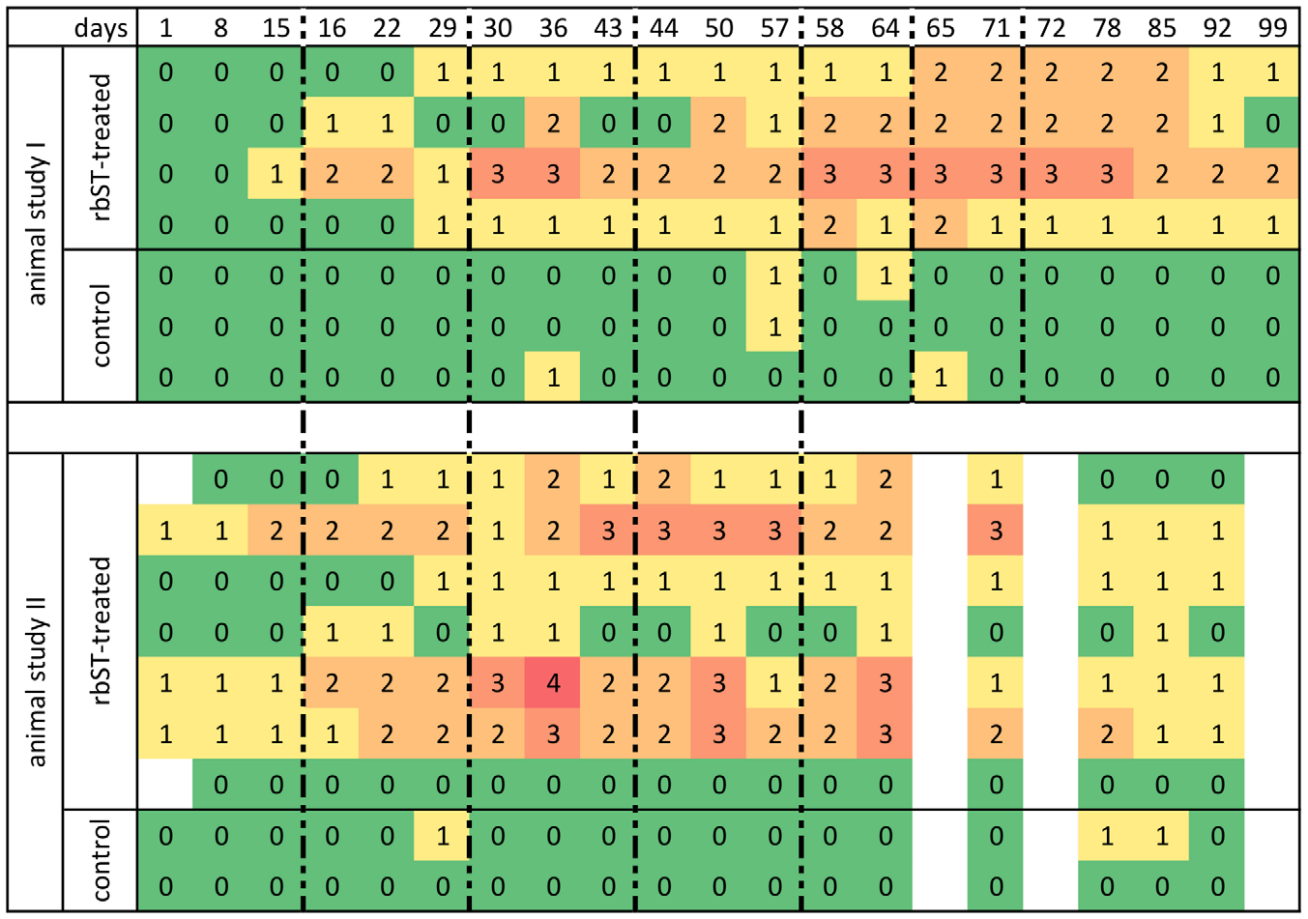
Number of biomarkers reacting above the respective decision limit. Results shown per cow (in animal studies I and II) and day of the controlled animal studies. Each row represents one individual cow. Vertical dotted lines indicate the treatment time points in both animal studies.

**Figure 5:**
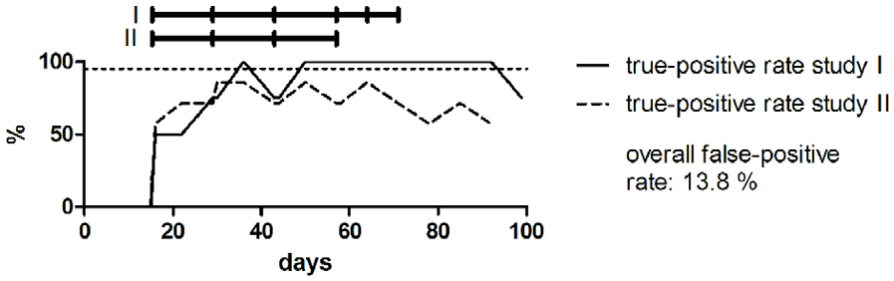
Predictive power (shown as true-positive and false-positive rates) of the additive biomarker analysis. True-positive rates were calculated for all samples from rbST-treated cows in their treatment and withdrawal periods of study I and II. False-positive rates were calculated for all samples from untreated cows from the two animal studies (adaptation period samples from all cows and all the samples from untreated cows). Samples were considered positive if one of the candidate biomarkers exceeded its respective decision limit. The treatment schedules of the two animal studies are indicated by black horizontal bars on top of the graph. The targeted 95% true-positive (<5% false-compliant) rate according to 2002/657/EC is indicated by the dotted horizontal line.

Since no single candidate biomarker was capable of predicting 95% of the rbST-treated samples correctly, we tested different possibilities of combining biomarker results for improvement of the predictive power of our 4-plex FCIA. One approach to do this is the additive biomarker analysis. In Figure 4, the number of candidate biomarkers responding above decision limit per cow and per time point within the animal studies is shown. As already described in paragraph 2 of the results section, there were big inter-individual differences: some cows responded in many markers, others only in one or two for some time points. There were also two extreme cases: one cow responded in all four tested markers above decision limit at one time point and another rbST-treated cow did not show any response above decision limit at any day. On the other hand, there were untreated cows, which showed positive responses in one of the candidate biomarkers. Figure 5 shows the true-positive rate obtained for the rbST-treated cows of both animal studies considering a sample positive, when at least one biomarker reacted above the respective decision limit. Although the true-positive rate obtained with the additive biomarker analysis was much higher than for the single candidate biomarkers, the 95% true-positive rate required for a screening method was only reached at some time points in study I within the biweekly treatment period. After the double rbST injection in study I, all of the cows were found positive, but this treatment frequency will not be found in real practice. Furthermore, also with the additive biomarker analysis, quite some false-positive results were obtained throughout the whole study.

### Statistical multiple biomarker analysis

**Figure 6:**
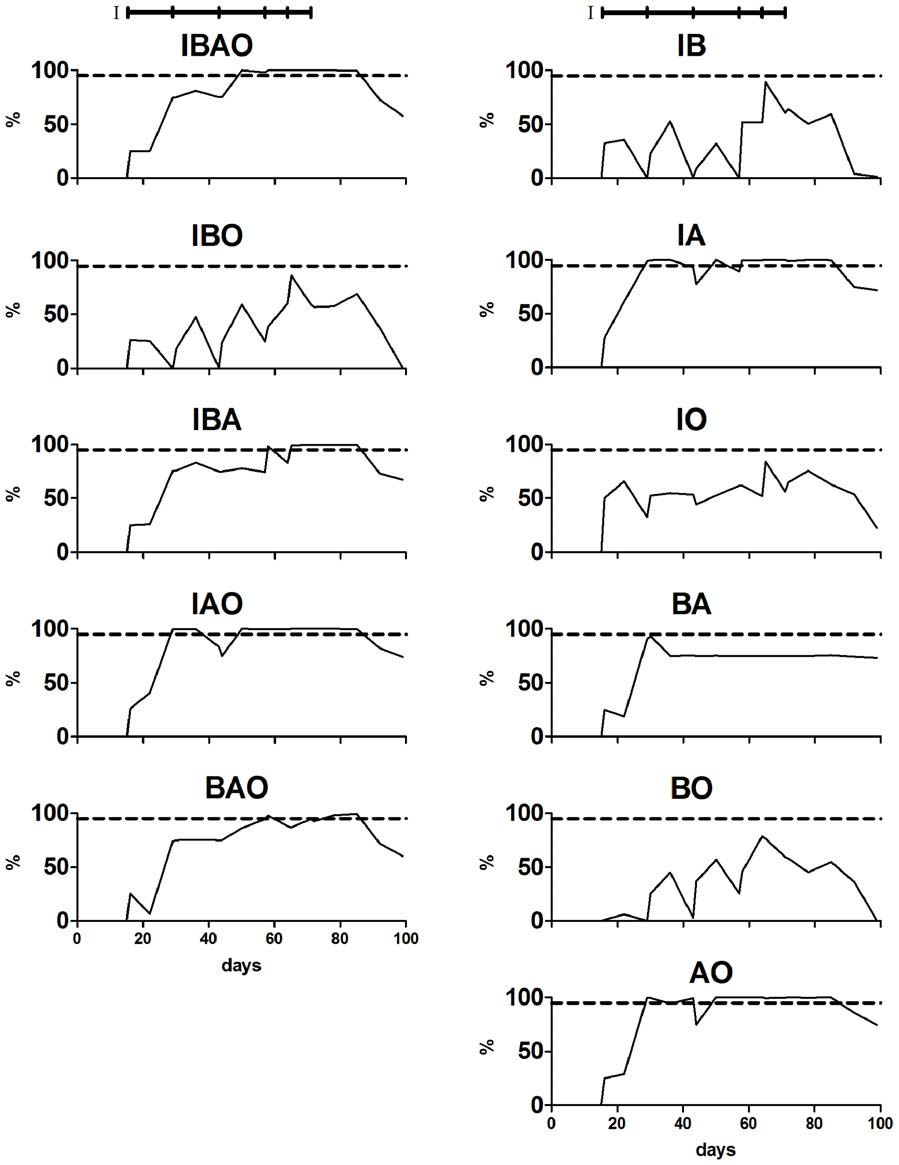
True-positive rates following statistical multiple biomarker analysis. True-positive rates, obtained with the prediction models based on the eleven different biomarker combinations, were calculated for rbST-treated cows from animal study I in their treatment and withdrawal period. The treatment schedules of animal study I is indicated by black horizontal bars on top of the graphs. The targeted 95% true-positive rate according to 2002/657/EC is indicated by the dotted horizontal lines.

Since the single biomarker analysis and additive biomarker analysis, which were both based on decision limits, did not deliver satisfying results for predicting rbST abuse, a different biomarker-combining approach was chosen for analysis of the data. K-nearest neighbours (kNN), a statistical prediction tool, was used to build a model from one group of data (Group A: all animals of animal study II and untreated animals from animal study I) and predict the results of Group B (rbST-treated cows of animal study I and 67 independent untreated cows) on basis of the built model. Eleven different models (one for every possible combination of two to four biomarkers) were evaluated to find the optimal biomarker combination for rbST abuse prediction. True-positive rates of Group B data were calculated for every biomarker combination over the time of the whole animal study and are shown in Figure 6 (Table S1 shows corresponding data). Six of the eleven different models yielded true-positive rates above the 95% true-positive rate required for a screening method at several time points. For the biomarker combinations IGF-1 - IGFBP2 - anti-rbST-antibodies (IBA) and IGFBP2 - anti-rbST-antibodies - osteocalcin (BAO), only one time point within the biweekly treatment period was above 95%. Note that in total, samples from eleven time points were obtained and analysed during the biweekly treatment period of animal study I. For the biomarker combinations IBAO and IA, four and six time points within the biweekly treatment period were above the 95% target respectively. Seven time points above the 95% target within the biweekly treatment period were reached by the prediction models based on the biomarker combinations IAO and AO. For the three best performing models (IA, AO and IAO), true-positive rates above 95% (<5% false-compliant) were reached following the second rbST injection. For IA, a true-prediction rate of almost 60% was observed already one week after the first rbST injection, whereas AO only showed 30%, which is in accordance with expectations since IGF-1 is a quick responding biomarker and osteocalcin has a delayed response time. Since all of the rbST-treated cows were detected by the three best performing models (IA, AO, IAO) at the end of the biweekly treatment period, no further increased prediction rate was observed due to the subsequent two weekly injections. After withdrawal of rbST, the true-positive rate of the models based on IA, AO and IAO remained above 95% for two more weeks and then declined to 70% four weeks after withdrawal.

Since we used all of the untreated animals of both animal studies for model building, false-positive rates for the eleven different models were calculated based on the results of the 67 independent untreated cows (Table S2). For the three best-performing prediction models IA, AO and IAO, false-positive rates ranged from 10.6% to 14.7%, which was quite acceptable, since samples screened positive must be analysed by a subsequent confirmatory analysis method according to Commission Decision 2002/657/EC anyway [23]. The confirmation method is based on the detection of an N-terminal peptide of somatotropin, which has a different terminal amino acid in the recombinant form of the hormone [26].

We concluded from the results of the here presented studies that the AO biomarker combination is the preferred model for predicting rbST abuse. It yielded seven out of eleven time points above the 95% target and if two biomarkers are equally well-suited for prediction as three biomarkers, the simpler version is favoured.

The results obtained proof that the developed 4-plex FCIA reduced to an AO biomarker combination 2-plex FCIA, applied to an *in vivo* evaluation and combined with a thorough statistical multiple biomarker analysis can detect more than 95% of the rbST-treated cows truly positive directly after the second rbST injection until the end of their treatment period and even thereafter. This meets the requirements of Commission Decision 2002/657/EC for a screening assay for the detection of banned veterinary drugs such as rbST [23].

When comparing with previously reported results of a 3-plex FCIA combining IGF-1, IGFBP2 and anti-rbST-antibodies [31], the models presented here seemingly perform somewhat less, especially at the beginning of the rbST treatment but the new models are much more realistic: Note that here, two completely independent groups were used for model building (Group A) and prediction (Group B), whereas in the 3-plex experiments [31], sample data used for prediction were from the same cows as the data on which the model was built, leading to an overestimation of true-positive results in that work.

## Discussion

For the first time, to the best of our knowledge, a 4-plex biomarker assay development and data evaluation model is presented for the detection of rbST abuse, which fulfils the requirements of Commission Decision 2002/657/EC for screening assays [23]. Furthermore, the extensive *in vivo* validation with two independent rbST animal treatment studies followed by statistical analysis revealed that a combination of just two candidate biomarkers is actually adequate for detection of rbST treatment. Therefore, even a 2-plex version (namely the combination of anti-rbST-antibodies and osteocalcin) of our assay would already be fit-for-purpose based on the data presented here.

Nevertheless some issues should be considered. First of all, for obvious ethical and cost reasons, the rbST treatment period was limited to 8 weeks in our animal studies, so we do not know yet how the prediction models would perform for long-term treated animals. As it can be seen in Figure 2C.1, the antibody biomarker response declined somewhat in course of the treatment period and we do not know whether this would influence the prediction quality in a prolonged treatment. Second, in the presented animal studies, cows were treated with rbST for the first time in their lives and there are no data about biomarker levels during a second treatment period after calving. According to the manufacturers' treatment schedule, dairy cows are treated starting from 9 weeks after calving until the end of the lactation (typically a biweekly treatment) and the following year again. Eppard *et al.* and Zwickl *et al.* reported that repeated treatment periods did not cause an immunological memory effect with enhanced antibody production in the second treatment period [33], [34]. For both situations, long-term treatment and repeated treatment, the IA, AO and IAO biomarker combinations should be tested and possibly the inclusion of other biomarkers could be considered. Since blood sampling in routine veterinary monitoring programmes might not be justified in some countries, we suggest a tiered approach according to Commission Decision 2002/657/EC. This would consist of three steps: First, a fast screening for anti-rbST-antibodies in tank milk using our previously described milk FCIA [29]. Second, in case of suspicious findings, a more detailed individual bovine serum biomarker profiling using the IA, AO or IAO FCIA presented here will provide additional evidence, since they are based on more biomarkers and data for individual cows. Note that in practice, a whole stable and not an individual cow is treated with rbST, thus increasing the chance of detecting rbST use. And third, for final confirmation of rbST itself in serum samples of suspect individual cows, a highly sensitive mass spectrometric confirmatory method, which fulfils the 2002/657/EC confirmatory method requirements, is to be used [26].

## Conclusions

In this study a multidisciplinary approach was used for the development of an *in vivo* validated screening assay for rbST abuse in dairy cows. Four candidate biomarkers for rbST abuse were assessed using a newly developed 4-plex flow cytometric immunoassay, *in vivo* validation studies and advanced statistics. Biomarkers indicative for rbST administration were evaluated based on two extensive animal studies with rbST-treated and untreated animals and an additional untreated reference population. Different data evaluation approaches were tested. The prediction tool kNN using a biomarker combination endogenously produced antibodies against rbST and osteocalcin proved to be very reliable and correctly predicted 95% of the treated samples starting from the second rbST injection until the end of the treatment period and even thereafter. This reduced 2-plex FCIA method (consisting of biomarkers anti-rbST antibodies and osteocalcin) combined with the statistical analysis approach was shown to be a fast, reliable and cost-effective approach to screen for rbST abuse in dairy cattle. These methods and models can be included in routine veterinary control programmes in the European Union for detection of rbST abuse and also in the control of rbST-free dairy farms in the United States of America and other countries.

## Materials and Methods

### Ethics statement

Permission for animal study I (EC2007/71) was obtained from the Ethical Commission of the Faculty of Veterinary Medicine of Ghent University (Belgium) on basis of the Dutch law on animal studies (Wet op de Dierproeven). For animal study II, permission (EC2010-21) was obtained from the Ethical Commission of the Animal Science Group of Wageningen University and Research Centre in Lelystad (The Netherlands).

### Chemicals and instruments

Ultrasonic bath, monosodium phosphate monohydrate (NaH_2_PO_4_×H_2_O), potassium dihydrogen phosphate (KH_2_PO_4_), sodium chloride (NaCl), sodium azide (NaN_3_) and Tween 20 were obtained from VWR International (Amsterdam, The Netherlands). Microcentrifuge Model 16K was purchased from Bio-Rad (Veenendaal, The Netherlands). Protein LoBind Tubes, Safe Lock Tubes (amber) and Centrifuge 5810R were obtained from Eppendorf (Hamburg, Germany). 1-Ethyl-3-(3-dimethylaminopropyl)carbodiimide (EDC), 2-(N-morpholino)ethanesulfonic acid (MES) hydrate, ovalbumin and bovine serum albumin (BSA) were obtained from Sigma-Aldrich (St. Louis, MO, USA). MultiScreen HTS filter plates were purchased from Millipore (Billerica, MA, USA). Purified bovine osteocalcin and mouse anti-bovine osteocalcin antibodies were obtained from Haematologic Technologies, Inc. (Essex Junction, VT, USA). Insulin-like growth factor-I (IGF-I; human recombinant) was purchased from Fitzgerald Industries International (North Acton, MA, USA). Insulin-like growth factor binding protein-2 (IGFBP-2; bovine recombinant, receptor grade) was purchased from IBT (Reutlingen, Germany). Mouse anti-IGF-1 was supplied by LifeSpan BioSciences, Inc. (clone SPM406, Seattle, WA, USA) and the rabbit anti-IGFBP-2 was from United States Biological (Swampscott, MA, USA). Monsanto rbST standard was obtained from the National Hormone & Peptide Program (NHPP) of Dr Parlow (Torrance, CA, USA). R-Phycoerythrin (PE)-labelled goat anti-bovine immunoglobulins (GAB-PE) were from Santa Cruz Biotechnology (Santa Cruz, CA, USA) and R-Phycoerythrin (PE)-labelled goat anti-mouse immunoglobulins (GAM-PE) and goat anti-rabbit immunoglobulins (GAR-PE) were purchased at Prozyme (San Leandro, CA, USA). Donor adult bovine serum was from HyClone (South Logan, UT, USA). Sodium hydroxide (NaOH), disodium hydrogen phosphate dihydrate (Na_2_HPO_4_×2 H_2_O) and hydrochloric acid (HCl) were purchased from Merck (Darmstadt, Germany). SeroMAP microspheres (microsphere sets 025, 050, 078 and 084) and sheath fluid were obtained from Luminex (Austin, TX, USA). The Luminex 100 IS 2.2 system consisting of a Luminex 100 analyser and a Luminex XY Platform was purchased from Applied Cytometry Systems (ACS, Dinnington, Sheffield, South Yorkshire, UK). Snijder Test tube rotator was from Omnilabo International (Breda, The Netherlands). 10 mL polypropylene tubes were obtained from Greiner Bio-One (Alphen aan de Rijn, The Netherlands). Glycine was purchased from Duchefa (Haarlem, The Netherlands) and sulfo-*N*-Hydroxysuccinimide (Sulfo-NHS) from Fluka (Buchs, Switzerland). Sodium dodecyl sulphate (SDS) was obtained from Serva (Heidelberg, Germany). The microtiter vari-shaker was purchased from Dynatech (Guernsey, UK). Posilac® (rbST) 500 mg single dose syringes and syringes with only the slow release formula were obtained from Monsanto Company (St. Louis, MO, USA) for animal study I and from Elanco Animal Health (Greenfield, IN, USA) for animal study II.

### Buffers and solutions

Buffers and solutions were prepared as follows: phosphate-buffered saline (PBS; 154 mM NaCl, 5.39 mM Na_2_HPO_4_, 1.29 mM KH_2_PO_4_, pH 7.4), PBST (PBS, 0.05% v/v Tween-20), PBSTB (0.1% w/v BSA in PBST), glycine solution I (GS I; 27.5 mM glycine, pH 0.5 adjusted with HCl), glycine solution II (GS II; 400 mM glycine, 0.3% w/v SDS, pH 10 adjusted with NaOH), MES buffer (50 mM, pH 5), blocking buffer (PBS, 0.1% w/v BSA, 0.02% v/v Tween-20, 0.05% w/v NaN_3_).

### Sample materials

Samples from different sources were used for analysis. Serum samples from two independent controlled animal treatment studies were used. In animal study I, eight Holstein cows were selected. These cows were all about 5 years old, divided in two groups of 4 animals each and treated with 500 mg rbST in slow-release formula or slow-release formula only. After a two-week adaptation period, they received an injection every second week, in total 4 times in accordance with the suggested treatment schedule by the manufacturer (http://www.fda.gov/downloads/AnimalVeterinary/Products/ApprovedAnimalDrugProducts/FOIADrugSummaries/ucm050022.pdf; accessed 2012 Apr 4). Since we did not know for sure whether we would see any response, the cows were thereafter treated two times more but with a weekly interval, followed by a final 4-week withdrawal period. In animal study II, 10 Holstein dairy cows were divided in two groups. In contrast to animal study I, these cows were of different age (2–8 years). After a 2-week adaptation period, 8 cows were treated every second week with 500 mg rbST in a slow-release formula during 8 weeks and 2 control cows were treated with the slow-release formula only. The biweekly treatment period according to manufacturers' guidelines was directly followed by a 4-week withdrawal period. In both studies, blood sampling was scheduled similarly: During the two week adaptation period, blood samples were collected weekly; during the treatment period, blood samples were collected a day before, a day after and a week after injection and during withdrawal, blood samples were collected weekly for four more weeks, which yielded 21 serum samples per cow in animal study I and 18 serum samples per cow in animal study II. The treatment schedule and blood sampling time points are shown in Figure S2. Unfortunately, one untreated cow died in the beginning of animal study I because of swollen hocks, which led to general inflammation and sepsis. Therefore in study I, results could be obtained for 4 rbST-treated and 3 untreated cows. Furthermore, one cow from animal study II fell sick (hock joint inflammation, lung infection and sepsis) in course of the experiment and its biomarker level results were excluded from statistical analysis. For investigation of natural physiological variations in biomarker levels, sera from 67 healthy, lactating cows varying in the age of two to eleven years, from two different locations, in different stages of their lactating cycle were analysed, to reflect a normal population of untreated dairy cows. Based on the origin of these animals the assumption of being untreated with rbST was justified.

### Standard preparation

Protein standards of IGF-1, IGFBP2 and osteocalcin, prepared in serum-matched buffer (80 mg mL^−1^ BSA in PBS), were used for standard curves ranging from 0.08 to 20 ng mL^−1^ for IGF-1 and osteocalcin and from 0.2 to 50 ng mL^−1^ for IGFBP2. Also blank standard samples (80 mg mL^−1^ BSA in PBS without any IGF-1, IGFBP2 and osteocalcin) were measured. Note that no standards are commercially available for anti-rbST-antibodies.

### Sample pre-treatment

A generic sample pre-treatment procedure which was crucial for removing non-specific interferences and making the candidate biomarkers accessible for detection was described previously [4], [28], [29], is depicted in Figure 1 and described in-depth in Materials S1.

### Microsphere preparation

Covalent coupling of 100 µg mL^−1^ Monsanto rbST standard, 100 µg mL^−1^ IGF-1 and 10 µg mL^−1^ IGFBP2 to seroMAP microspheres (sets 050, 025, 078 respectively) was described before [4], [28], [29]. Coupling 75 µg mL^−1^ osteocalcin to microspheres (set 084) was done following the same procedure.

### Four-plex flow cytometric immunoassay procedure

The assay procedure for detection of three biomarkers was described before [31] and is similar for four biomarkers in the present study and summarized in Figure 1. The samples were analysed in duplicate in the flow cytometer at 1 µL s^−1^ until 50 microspheres per set were counted, up to a maximum of 50 µL per sample. A typical analysis of a full 96 well microtiter plate takes 3.5 hours starting from raw serum until the results are obtained.

### Data analysis

Raw median fluorescence intensities (MFIs) were measured by the flow cytometer for every single candidate biomarker. Every sample was measured in duplicate and MFIs were averaged before further analysis. For IGF-1, IGFBP2 and osteocalcin, B/B0 values were calculated per sample by dividing the measured MFI by the MFI of a blank biomarker-free standard. Then, concentrations were recalculated from standard curves (non-linear four-parameter curve fit) using GraphPad Prism program (GraphPad Software Inc., San Diego, USA) for IGF-1 and osteocalcin. For IGFBP2, no complete inhibition could be obtained with the available standard protein, therefore, no actual concentrations were determined and B/B0 values were simply used. For anti-rbST-antibodies, which are endogenously produced by the cow in response to rbST treatment, no standard was available. To be able to normalize, measured sample MFIs were divided by the MFI of one serum sample, which was measured every time (B/Bd). This serum was donor adult bovine serum which was a mixture of sera from many cows from one herd. Since this is produced in large amounts, it can be used for a long time with constant quality.

To assess the 4-plex FCIA quality and compare it to other methods, assay performance characteristics were calculated, such as IC_50_, inter-assay and intra-assay variation (describing precision and ruggedness). For IGF-1, IGFBP2 and osteocalcin, IC_50_ was read from standard curves at 50% inhibition of the signal of the blank. For all candidate biomarkers, inter-assay variation was determined by measuring 8 different serum samples on 8 days. Mean of results (concentrations for IGF-1 and osteocalcin, B/B0 for IGFBP2 and B/Bd for anti-rbST-antibodies), standard deviation and percentaged standard deviation (%CV) were calculated for every serum. The average of the 8 percentaged standard deviations was the inter-assay deviation. Intra-assay variation was calculated the same way from 8 repetitions of 8 sera within one microtiter plate.

#### Single biomarker analysis approach

Using a single biomarker for prediction of unknown samples as rbST-treated or untreated, the calculation of decision limits for each biomarker was necessary. These were based on the results obtained from a population of 67 untreated dairy cows being diverse in age, in lactation stage and in origin. For every biomarker, results were averaged and two-times the standard deviation was added to obtain the decision limit. Samples found to show concentrations (for IGF-1 and osteocalcin), B/B0 (for IGFBP2) or B/Bd (for anti-rbST antibodies) beyond the respective calculated decision limit, were considered as rbST-treated (positive). True-positive and false-positive rates could be calculated for every single biomarker from the results of the controlled animal studies.

#### Additive biomarker analysis

After evaluating biomarker profiles and true-positive rates based on single biomarkers, an additive biomarker approach was tested. Here, a sample was considered as rbST-treated when at least one of the candidate biomarkers reacted above decision limit and also here, true-positive and false-positive rates were calculated.

#### Multiple biomarker statistical approach

After evaluating single candidate biomarkers and testing the additive biomarker approach, we assessed how well a statistical combination of two to four markers was capable to predict rbST abuse. Therefore, a k-nearest neighbours prediction model (kNN) in the R environment [42] and functions available in R package e1071 [43] were used to evaluate all eleven theoretical combinations of two to four biomarkers. As in the single biomarker approach, recalculated concentrations for IGF-1 and osteocalcin and B/B0 signals for IGFBP2 as well as B/Bd signals for rbST-induced antibodies for every sample from the animal studies were included in the data analysis. For obvious ethical reasons, we had only a limited number of rbST-treated animals available. Therefore, all serum sample time points per cow (21 time points in the trial period of 14 weeks for animal study I and 18 time points in the trial period of 13 weeks for animal study II) were used for data analysis, despite the fact that these were not completely independent. However, only data from independent cows were used for model building and sample prediction.

First, the whole data set was divided into two groups: Group A data were used to build the time-point-independent prediction model. Therefore and to use sufficient sample numbers for the model building, this group contained all data from animal study II (diverse population with biweekly treatment only). Furthermore, since two control animals were not enough to represent untreated cows, Group A also contained the data from the untreated animals of animal study I. In total, 98 samples from treated and 119 samples from untreated cows were used for model building. Group B data were used for prediction based on the Group A model. Group B contained the data from the rbST-treated cows of animal study I (uniform in age with biweekly treatment and two additional weekly injections) and the 67 untreated cows. Note that these are sample data independent from Group A data.

For model building of the Group A data, a training and test set were chosen by using a stratified repeated random sub-sampling approach, which means that 70% of the rbST-treated and 70% of the untreated samples were selected for the training set and the remaining 30% of both groups for the test set for internal validation, which is necessary to build a strong model. Subsequently, concentrations, B/B0 and B/Bd values of the training set were auto-scaled and a kNN model was built on the training set data. The optimal number of k (1≤k≤10) was chosen based on the bootstrapping approach [44] leaving out 10% of the training data (randomly with replacement), which was repeated 10-times. The resulting model was validated with the test set data and thereafter used for predicting Group B data. To obtain an average performance of the model, this procedure was run 10,000 times; every time different randomly chosen training and test sets of Group A data were applied. Correctly and falsely predicted results were evaluated for Group B and a true-positive rate and false-positive rate could be calculated for every Group B sample.

## Supporting Information

Figure S1 **Standard curves of the three rbST-dependent biomarkers IGF-1, IGFBP2 and osteocalcin.** Each data point is the mean of 8 separate measurements in a serum-matched buffer (80 mg mL^−1^ BSA in PBS solution). All curves relate to 80-times diluted sera.
(TIF)

Figure S2 **Treatment schedule and sampling time points for animal studies I and II.** Arrows indicate the treatment of the cows with rbST in slow-release formula or the slow-release formula only; bold vertical lines indicate blood sampling time points.
(TIF)

Materials S1 **Serum preparation, generic serum pre-treatment and 4-plex FCIA for serum candidate biomarkers.**
(DOCX)

Table S1 **True-positive rates of the statistical multiple biomarker analysis.** True-positive rates, obtained with the prediction models based on the eleven different biomarker combinations, were calculated for rbST-treated cows from animal study I in their treatment (day 16–71) and withdrawal period (day 72–99).
(DOCX)

Table S2 **False-positive rates of the statistical multiple biomarker analysis.** Results were calculated for 67 independent untreated cows predicted with the eleven different biomarker combination models.
(DOCX)

## References

[1] Teale P, Barton C, Driver PM, Kay RG (2009) Biomarkers: unrealized potential in sports doping analysis. Bioanalysis 1: 1103–1118. 21083078 10.4155/bio.09.87

[2] Cacciatore G, Eisenberg SW, Situ C, Mooney MH, Delahaut P, et al (2009) Effect of growth-promoting 17[beta]-estradiol, 19-nortestosterone and dexamethasone on circulating levels of nine potential biomarker candidates in veal calves. Anal Chim Acta 637: 351–359. 19286051 10.1016/j.aca.2008.11.027

[3] Draisci R, Montesissa C, Santamaria B, D'Ambrosio C, Ferretti G, et al (2007) Integrated analytical approach in veal calves administered the anabolic androgenic steroids boldenone and boldione: urine and plasma kinetic profile and changes in plasma protein expression. Proteomics 7: 3184–3193. 17676661 10.1002/pmic.200601039

[4] Smits NGE, Bremer MGEG, Ludwig SKJ, Nielen MWF (2012) Development of a flow cytometric immunoassay for recombinant bovine somatotropin-induced antibodies in serum of dairy cows. Drug Test Analysis 4: 362–367. 10.1002/dta.336 21964757

[5] Chung L, Baxter RC (2009) Detection of growth hormone responsive proteins using SELDI–TOF mass spectrometry. Growth Horm IGF Res 19: 383–387. 19467616 10.1016/j.ghir.2009.04.019

[6] Chung L, Clifford D, Buckley M, Baxter RC (2006) Novel biomarkers of human growth hormone action from serum proteomic profiling using protein chip mass spectrometry. J Clin Endocrinol Metab 91: 671–677. 16303837 10.1210/jc.2005-1137

[7] Ding J, List EO, Okada S, Kopchick JJ (2009) Perspective: Proteomic approach to detect biomarkers of human growth hormone. Growth Horm IGF Res 19: 399–407. 19501004 10.1016/j.ghir.2009.04.018 PMC2760539

[8] Ding J, Okada S, Jørgensen JOL, Kopchick JJ (2011) Novel serum protein biomarkers indicative of growth hormone doping in healthy human subjects. Proteomics 11: 3565–3571. 21751372 10.1002/pmic.201100077 PMC3517138

[9] Boateng J, Kay R, Lancashire L, Brown P, Velloso C, et al (2009) A proteomic approach combining MS and bioinformatic analysis for the detection and identification of biomarkers of administration of exogenous human growth hormone in humans. Proteom Clin Appl 3: 912–922. 10.1002/prca.200800190 21136995

[10] Mooney MH, Situ C, Cacciatore G, Hutchinson T, Elliott C, et al (2008) Plasma biomarker profiling in the detection of growth promoter use in calves. Biomarkers 13: 246–256. 18415798 10.1080/13547500701838593

[11] Powrie JK, Bassett EE, Rosen T, Jørgensen JO, Napoli R, et al (2007) Detection of growth hormone abuse in sport. Growth Horm IGF Res 17: 220–226. 17339122 10.1016/j.ghir.2007.01.011

[12] Kniess A, Ziegler E, Kratzsch J, Thieme D, Müller R (2003) Potential parameters for the detection of hGH doping. Anal Bioanal Chem 376: 696–700. 12750868 10.1007/s00216-003-1926-x

[13] Longobardi S, Keay N, Ehrnborg C, Cittadini A, Rosen T, et al (2000) Growth hormone (GH) effects on bone and collagen turnover in healthy adults and its potential as a marker of GH abuse in sports: A double blind, placebo-controlled study. J Clin Endocrinol Metab 85: 1505–1512. 10770189 10.1210/jcem.85.4.6551

[14] Dall R, Longobardi S, Ehrnborg C, Keay N, Rosén T, et al (2000) The effect of four weeks of supraphysiological growth hormone administration on the insulin-like growth factor axis in women and men. J Clin Endocr Metab 85: 4193–4200. 11095453 10.1210/jcem.85.11.6964

[15] Erotokritou-Mulligan I, Eryl Bassett E, Cowan DA, Bartlett C, Milward P, et al (2010) The use of growth hormone (GH)-dependent markers in the detection of GH abuse in sport: Physiological intra-individual variation of IGF-I, type 3 pro-collagen (P-III-P) and the GH-2000 detection score. Clin Endocrinol 72: 520–526. 10.1111/j.1365-2265.2009.03668.x 19650783

[16] Healy M-L, Dall R, Gibney J, Bassett E, Ehrnborg C, et al (2005) Toward the development of a test for growth hormone (GH) abuse: A study of extreme physiological ranges of GH-dependent markers in 813 elite athletes in the postcompetition setting. J Clin Endocr Metab 90: 641–649. 15546908 10.1210/jc.2004-0386

[17] Sartorio A, Jubeau M, Agosti F, Marazzi N, Rigamonti A, et al (2006) A follow-up of GH-dependent biomarkers during a 6-month period of the sporting season of male and female athletes. J Endocrinol Invest 29: 237–243. 16682837 10.1007/BF03345546

[18] Wallace JD, Cuneo RC, Lundberg PA, Rosen T, Jorgensen JOL, et al (2000) Responses of markers of bone and collagen turnover to exercise, growth hormone (GH) administration, and GH withdrawal in trained adult males. J Clin Endocrinol Metab 85: 124–133. 10634375 10.1210/jcem.85.1.6262

[19] Castigliego L, Grifoni G, Rosati R, Iannone G, Armani A, et al (2009) On the alterations in serum concentration of somatotropin and insuline-like growth factor 1 in lactating cows after the treatment with a little studied recombinant bovine somatotropin. Res Vet Sci 87: 29–35. 19062055 10.1016/j.rvsc.2008.10.012

[20] Zhao X, McBride BW, Trouten-Radford LM, Golfman L, Burton JH (1994) Somatotropin and insulin-like growth factor-I concentrations in plasma and milk after daily or sustained-release exogenous somatotropin administrations. Domest Anim Endocrin 11: 209–216. 10.1016/0739-7240(94)90028-0 8045102

[21] Food and Drug Administration (1993) Animal Drugs, Feeds, and Related Products; Sterile Sometribove Zinc Suspension. 58 Federal Register 59946.

[22] European Union (1999) Council Decision 1999/879/EC of 17 December 1999 concerning the placing on the market and administration of bovine somatotrophin (BST) and repealing Decision 90/218/EEC. Off J Eur Commun L 331: 71–72.

[23] European Union (2002) Commission Decision 2002/657/EC of 12 August 2002 implementing Council Directive 96/23/EC concerning the performance of analytical methods and the interpretation of results. Off J Eur Commun L 221: 8–36.

[24] Castigliego L, Iannone G, Grifoni G, Rosati R, Gianfaldoni D, et al (2007) Natural and recombinant bovine somatotropin: immunodetection with a sandwich ELISA. J Dairy Res 74: 79–85. 16978434 10.1017/S0022029906002159

[25] Lucy MC, Byatt JC, Curran TL, Curran DF, Collier RJ (1994) Placental lactogen and somatotropin: hormone binding to the corpus luteum and effects on the growth and functions of the ovary in heifers. Biol Reprod 50: 1136–1144. 8025170 10.1095/biolreprod50.5.1136

[26] Le Breton MH, Rochereau-Roulet S, Chereau S, Pinel G, Delatour T, et al (2010) Identification of cows treated with recombinant bovine somatotropin. J Agr Food Chem 58: 729–733. 20041652 10.1021/jf903032q

[27] Le Breton MH, Rochereau-Roulet S, Pinel G, Cesbron N, Le Bizec B (2009) Elimination kinetic of recombinant somatotropin in bovine. Anal Chim Acta 637: 121–127. 19286020 10.1016/j.aca.2008.09.003

[28] Bremer MGEG, Smits NGE, Haasnoot W, Nielen MWF (2010) Multiplex ready flow cytometric immunoassay for total insulin like growth factor 1 in serum of cattle. Analyst 135: 1147–1152. 20419268 10.1039/b925372f

[29] Ludwig SKJ, Smits NGE, Bremer MGEG, Nielen MWF (2012) Monitoring milk for antibodies against recombinant bovine somatotropin using a microsphere immunoassay-based biomarker approach. Food Control 26: 68–72.

[30] Rochereau-Roulet S, Gaudin I, Chéreau S, Prévost S, André-Fontaine G, et al (2011) Development and validation of an enzyme-linked immunosorbent assay for the detection of circulating antibodies raised against growth hormone as a consequence of rbST treatment in cows. Anal Chim Acta 700: 189–193. 21742132 10.1016/j.aca.2011.01.035

[31] Smits NGE, Ludwig SKJ, Van der Veer G, Bremer MGEG, Nielen MWF (2012) Serum biomarker profiling for detection of recombinant bovine somatotropin abuse using a multiplex flow cytometric immunoassay. Analyst doi: 10.1039/c1032an35226e. 10.1039/c2an35226e 22805655

[32] Kicman AT, Miell JP, Teale JD, Powrie J, Wood PJ, et al (1997) Serum IGF-I and IGF binding proteins 2 and 3 as potential markers of doping with human GH. Clin Endocrinol 47: 43–50. 10.1046/j.1365-2265.1997.2111036.x 9302371

[33] Eppard PJ, Rogan GJ, Boysen BG, Miller MA, Hintz RL, et al (1992) Effect of high doses of a sustained-release bovine somatotropin on antibody formation in dairy cows. J Dairy Sci 75: 2959–2967. 1460127 10.3168/jds.S0022-0302(92)78059-X

[34] Zwickl CM, Smith HW, Tamura RN, Bick PH (1990) Somatotropin antibody formation in cows treated with a recombinant bovine somatotropin over two lactations. J Dairy Sci 73: 2888–2895. 2283416 10.3168/jds.S0022-0302(90)78976-X

[35] Lee AJ, Hodges S, Eastell R (2000) Measurement of osteocalcin. Ann Clin Biochem 37: 432–446. 10902858 10.1177/000456320003700402

[36] Armstrong JD, Cohick WS, Harvey RW, Heimer EP, Campbell RM (1993) Effect of feed restriction on serum somatotropin, insulin-like growth factor-I-(IGF-I) and IGF binding proteins in cyclic heifers actively immunized against growth hormone releasing factor. Domest Anim Endocrin 10: 315–324. 10.1016/0739-7240(93)90035-a 7508357

[37] Blum WF, Horn N, Kratzsch J, Jørgensen JO, Juul A, et al (1993) Clinical studies of IGFBP-2 by radioimmunoassay. Growth Regulat 3: 100–104. 7683512

[38] Friedrich N, Alte D, Völzke H, Spilcke-Liss E, Lüdemann J, et al (2008) Reference ranges of serum IGF-1 and IGFBP-3 levels in a general adult population: Results of the Study of Health in Pomerania (SHIP). Growth Horm IGF Res 18: 228–237. 17997337 10.1016/j.ghir.2007.09.005

[39] Sato R, Onda K, Ochiai H, Iriki T, Yamazaki Y, et al (2011) Serum osteocalcin in dairy cows: Age-related changes and periparturient variation. Res Vet Sci 91: 196–198. 21300389 10.1016/j.rvsc.2010.12.007

[40] Kerr DE, Laarveld B, Fehr MI, Manns JG (1991) Profiles of serum IGF-I concentrations in calves from birth to eighteen months of age and in cows throughout the lactation cycle. Can J Anim Sci 71: 695–705.

[41] Sharma BK, Vandehaar MJ, Ames NK (1994) Expression of insulin-like growth factor-I in cows at different stages of lactation and in late lactation cows treated with somatotropin. J Dairy Sci 77: 2232–2241. 7525673 10.3168/jds.S0022-0302(94)77166-6

[42] R Development Core Team (2011) R: A language and environment for statistical computing.

[43] Dimitriadou E, Hornik K, Leisch F, Meyer D, Weingessel A (2011) Misc functions of the department of statistics (e1071), TU Wien.

[44] Efron B (1979) Bootstrap methods: Another look at the jackknife. Ann Stat 7: 1–26.

[45] Nelson AE, Ho KKY, Ghigo E, Lanfranco F, Strasburger CJ (2011) Detection of growth hormone doping in sport using growth hormone-responsive markers. In: Melmed S, editor. Hormone use and abuse by athletes: Springer US. pp. 139–150.

[46] Di Luigi L, Rigamonti AE, Agosti F, Mencarelli M, Sgrò P, et al (2009) Combined evaluation of resting IGF1, N-terminal propeptide of type III procollagen and C-terminal cross-linked telopeptide of type I collagen levels might be useful for detecting inappropriate GH administration in female athletes. Eur J Endocrinol 160: 753–758. 19258430 10.1530/EJE-08-0884

[47] McGuire MA, Vicini JL, Bauman DE, Veenhuizen JJ (1992) Insulin-like growth factors and binding proteins in ruminants and their nutritional regulation. J Anim Sci 70: 2901–2910. 1383181 10.2527/1992.7092901x

