# Supplementary figures and images for: Multiple Protein Biomarker Assessment for Recombinant Bovine Somatotropin (rbST) Abuse in Cattle
Source: PLoS One. 2012 Dec 27;7(12):e52917. doi: 10.1371/journal.pone.0052917 (PMC3531382; doi:10.1371/journal.pone.0052917)

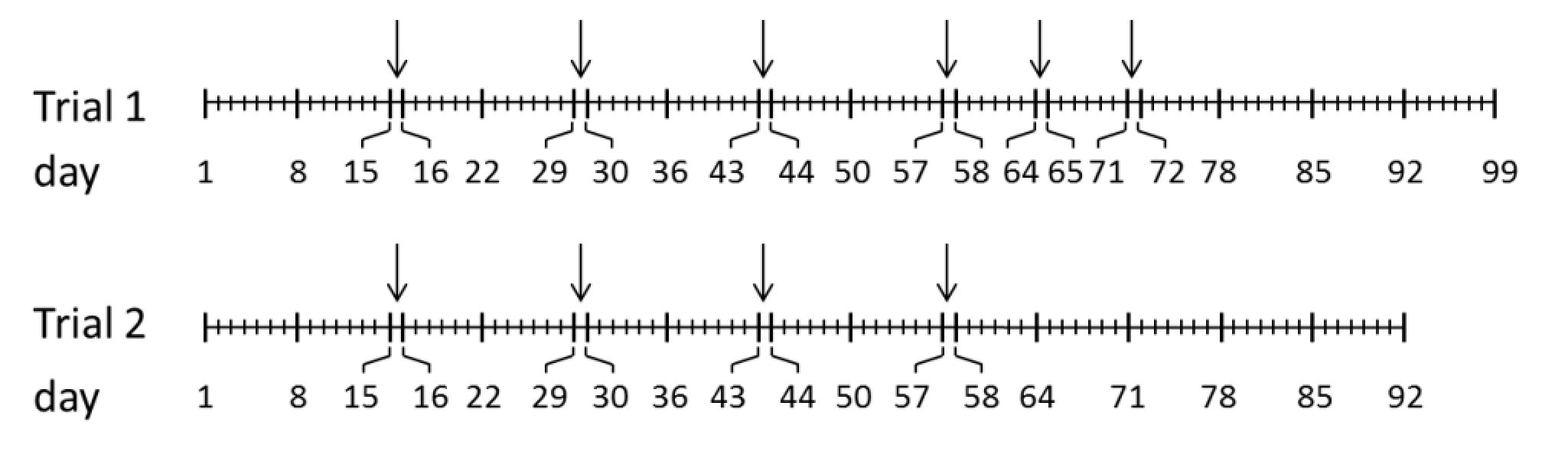

Supplement: Figure S2 — Treatment schedule and sampling time points for animal studies I and II. Arrows indicate the treatment of the cows with rbST in slow-release formula or the slow-release formula only; bold vertical lines indicate blood sampling time points. (TIF) [file pone.0052917.s002.tif]
