# Supplementary material for: Multiple Protein Biomarker Assessment for Recombinant Bovine Somatotropin (rbST) Abuse in Cattle
Source: PLoS One. 2012 Dec 27;7(12):e52917. doi: 10.1371/journal.pone.0052917 (PMC3531382; doi:10.1371/journal.pone.0052917)
Supplement: Materials S1 — Serum preparation, generic serum pre-treatment and 4-plex FCIA for serum candidate biomarkers. (DOCX) [file pone.0052917.s003.docx]

# Supporting information material

## Detailed work flow description

### Serum preparation

After blood collection, blood samples were kept at room temperature for 4 h to coagulate. After coagulation, samples were centrifuged for 10 min at 3,000 g, and sera were collected. Serum samples were stored at -80 ºC.

### Generic serum pretreatment

For the generic serum pretreatment procedure 25 µl glycine solution I (27.5 mM glycine pH 0.5) were added to 25 µl serum or standard sample in a polypropylene tube under constant vortexing. After 60 minutes incubation at room temperature, 50 µl glycine solution II (400 mM Glycine, 0.3% m/v SDS, pH 10) were added under constant vortexing and samples were further diluted by addition of 1.9 mL 0.1 % BSA in PBST (in total 80-times diluted).

### Four-plex flow cytometric immunoassay procedure

For the 4-plex FCIA procedure, 10 µl primary antibody mixture (1:625 mouse anti-IGF-1, 1:25,000 rabbit anti-IGFBP2, 1:100,000 mouse anti-osteocalcin) were added to 100 µl pretreated and diluted sample in a filter bottom microtiter plate and incubated at 4 °C under orbital shaking for 15 minutes. Thereafter, 10 µl microsphere mixture suspension (containing approximately 1250 microspheres per microsphere set) were added to each well and incubated at 4 °C for one hour under orbital shaking. After centrifugation at 130 g for one minute, everything not bound to the microspheres was removed from the well. For washing, 200 µl PBST were added and the filter bottom microtiter plate was centrifuged again at 130 g for one minute. Then, 125 µl PE-antibody mixture (1:625 goat anti-mouse PE, 1:1,000 goat-anti rabbit PE, 1:1,000 goat anti-bovine PE) were added to each well and the plate was incubated for 30 minutes at 4 °C under orbital shaking. Then, the plate was centrifuged at 130 g for one minute and 125 µl PBST were added. Thereafter, the plate was put into the LX-100 flow cytometer for measurement. Microspheres from every sample were analysed in a flow of 1 µl s^-1^ until 50 microspheres per set were counted up to a maximum of 50 µL per sample. Each microsphere set was identified by its unique colour by a red laser and the fluorescence intensity of the PE attached to each microsphere was measured by a green laser. Median fluorescence intensities were obtained from every analysed sample.
