# Supplementary material for: Multiple Protein Biomarker Assessment for Recombinant Bovine Somatotropin (rbST) Abuse in Cattle
Source: PLoS One. 2012 Dec 27;7(12):e52917. doi: 10.1371/journal.pone.0052917 (PMC3531382; doi:10.1371/journal.pone.0052917)
Supplement: Table S1 — True-positive rates of the statistical multiple biomarker analysis. True-positive rates, obtained with the prediction models based on the eleven different biomarker combinations, were calculated for rbST-treated cows from animal study I in their treatment (day 16–71) and withdrawal period (day 72–99). (DOCX) [file pone.0052917.s004.docx]

| **biomarker combinations** | **IBAO** | **IBO** | **IBA** | **IAO** | **BAO** | **IB** | **IA** | **IO** | **BA** | **BO** | **AO** |
| --- | --- | --- | --- | --- | --- | --- | --- | --- | --- | --- | --- |
| **days** | **true-positive rate [%]** | | | | | | | | | | |
| 1 | 0 | 0 | 0 | 0 | 0 | 0 | 0 | 0 | 0 | 0 | 0 |
| 8 | 0 | 0 | 0 | 0 | 0 | 0 | 0 | 0 | 0 | 0 | 0 |
| 15 | 0 | 0 | 0 | 0 | 0 | 0 | 0 | 0 | 0 | 0 | 0 |
| 16 | 25.0 | 26.4 | 25.0 | 25.8 | 25.1 | 32.2 | 27.7 | 50.2 | 25.0 | 1.0 | 25.0 |
| 22 | 25.1 | 25.4 | 25.9 | 40.6 | 6.5 | 35.6 | 61.4 | 65.8 | 18.6 | 6.3 | 28.9 |
| 29 | 74.5 | 0.0 | 75.3 | 99.7 | 73.8 | 0.0 | 99.1 | 32.6 | 90.4 | 0.3 | 99.8 |
| 30 | 75.1 | 18.3 | 75.9 | 99.7 | 75.0 | 22.9 | 100.0 | 52.5 | 93.5 | 25.4 | 99.8 |
| 36 | 81.2 | 47.6 | 83.2 | 99.9 | 75.3 | 52.7 | 100.0 | 54.7 | 75.0 | 44.9 | 94.3 |
| 43 | 75.0 | 0.4 | 75.0 | 83.5 | 75.0 | 0.5 | 93.5 | 53.3 | 75.3 | 3.0 | 99.6 |
| 44 | 75.0 | 24.0 | 75.0 | 75.0 | 75.0 | 8.9 | 77.7 | 44.4 | 75.0 | 37.0 | 75.0 |
| 50 | 100.0 | 59.2 | 78.0 | 100.0 | 86.0 | 32.0 | 100.0 | 52.8 | 75.2 | 56.8 | 100.0 |
| 57 | 98.1 | 25.1 | 74.6 | 99.9 | 94.4 | 0.1 | 89.4 | 61.6 | 74.7 | 25.7 | 100.0 |
| 58 | 99.9 | 38.7 | 98.4 | 100.0 | 97.8 | 51.9 | 100.0 | 61.5 | 75.0 | 46.0 | 100.0 |
| 64 | 100.0 | 60.3 | 83.0 | 100.0 | 87.8 | 51.7 | 99.9 | 52.0 | 75.0 | 78.6 | 100.0 |
| 65 | 100.0 | 86.1 | 99.3 | 100.0 | 86.9 | 89.2 | 100.0 | 84.2 | 75.0 | 76.4 | 99.6 |
| 71 | 100.0 | 59.2 | 100.0 | 100.0 | 94.6 | 60.6 | 100.0 | 56.2 | 74.7 | 59.4 | 100.0 |
| 72 | 100.0 | 56.9 | 100.0 | 100.0 | 92.5 | 63.9 | 99.1 | 65.0 | 75.0 | 58.0 | 100.0 |
| 78 | 99.9 | 57.5 | 100.0 | 100.0 | 98.1 | 50.4 | 100.0 | 75.7 | 75.0 | 45.2 | 100.0 |
| 85 | 99.6 | 69.0 | 100.0 | 100.0 | 99.5 | 59.4 | 100.0 | 62.8 | 75.5 | 54.7 | 100.0 |
| 92 | 72.6 | 37.5 | 73.2 | 82.0 | 71.7 | 4.0 | 74.8 | 53.6 | 74.1 | 36.2 | 85.8 |
| 99 | 57.8 | 0.1 | 67.3 | 74.0 | 60.3 | 1.5 | 71.8 | 22.3 | 73.2 | 0.3 | 74.8 |
