# Supplementary material for: Multiple Protein Biomarker Assessment for Recombinant Bovine Somatotropin (rbST) Abuse in Cattle
Source: PLoS One. 2012 Dec 27;7(12):e52917. doi: 10.1371/journal.pone.0052917 (PMC3531382; doi:10.1371/journal.pone.0052917)
Supplement: Table S2 — False-positive rates of the statistical multiple biomarker analysis. Results were calculated for 67 independent untreated cows predicted with the eleven different biomarker combination models. (DOCX) [file pone.0052917.s005.docx]

| **biomarker combination** | **IBAO** | **IBO** | **IBA** | **IAO** | **BAO** | **IB** | **IA** | **IO** | **BA** | **BO** | **AO** |
| --- | --- | --- | --- | --- | --- | --- | --- | --- | --- | --- | --- |
|  | **false-positive rate [%]** | | | | | | | | | | |
| 67 untreated cows | 5.5 | 9.7 | 6.6 | 10.6 | 8.8 | 25 | 14.7 | 24.6 | 13.4 | 11.7 | 11.8 |
